# Experiences and Hopes Among Patients with Colorectal Carcinoma and Peritoneal Metastases Who Are Participating in an Early-Phase Clinical Trial

**DOI:** 10.3390/cancers18020244

**Published:** 2026-01-13

**Authors:** Lena Fauske, Øyvind S. Bruland, Anne Holtermann, Stein G. Larsen

**Affiliations:** 1 Department of Oncology, Norwegian Radium Hospital, Oslo University Hospital, 0379 Oslo, Norway; osb@ous-hf.no; 2 Faculty of Medicine, Institute for Clinical Medicine, University of Oslo, 0318 Oslo, Norway; 3 Department of Oncological Surgery, Norwegian Radium Hospital, Oslo University Hospital, 0379 Oslo, Norway; holtea@ous-hf.no (A.H.); stl@ous-hf.no (S.G.L.)

**Keywords:** clinical trial, peritoneal metastasis, cytoreductive surgery, hyperthermic intraperitoneal chemotherapy, radiopharmaceutical therapy, qualitative methodology, patient perspectives

## Abstract

*Background:* Radspherin^®^ is a novel α-emitting radiopharmaceutical administered intraperitoneally following complete cytoreductive surgery and hyperthermic intraperitoneal chemotherapy (CRS-HIPEC) for peritoneal metastases. It delivers short-range radiation aimed at eliminating residual microscopic disease. This qualitative study explored how participants with colorectal cancer experienced participating in an early-phase clinical trial involving CRS-HIPEC followed by Radspherin^®^. *Materials and Methods:* Semi-structured interviews were conducted with ten participants enrolled in a phase 1/2a trial involving CRS-HIPEC and intraperitoneal Radspherin^®^. The analysis was guided by a phenomenological and interpretive approach using reflexive thematic analysis. *Results:* Participants expressed a strong sense of motivation and hope tied specifically to receiving Radspherin^®^, which they perceived as an opportunity to improve their prognosis. Many also viewed participation as a contribution to future cancer research. None attributed complications or side effects to Radspherin^®^. Clear and supportive verbal communication from healthcare professionals was highly valued, while the written information was described as overwhelming. Despite fears of recurrence, most participants remained optimistic about regaining a meaningful life. While experiences with Radspherin^®^ were largely positive, participants also described pain, fatigue, and prolonged recovery related to CRS-HIPEC, including ongoing functional and psychosocial challenges. *Conclusions:* Participants associated Radspherin^®^ with hope and a therapeutic benefit but did not link it to their adverse events. Their willingness to participate in experimental treatment was shaped by trust in clinicians, clear communication, and a desire for extended survival. However, the burden of CRS-HIPEC-related side effects underscores the importance of tailored follow-up and support.

## Simple Summary

This study explored how participants with peritoneal metastases from colorectal cancer experienced receiving an experimental treatment called Radspherin^®^ two days after having completed cytoreductive surgery and heated intraperitoneal chemotherapy (CRS-HIPEC). Radspherin^®^ is a novel radionuclide treatment injected directly into the abdominal cavity aiming to eradicate remaining cancer cells. Participants who took part in this early-phase clinical trial were interviewed about their experiences. They felt hopeful and motivated by the chance to receive Radspherin^®^, which they saw as a potential way to further improve their outcome. None attributed side effects to Radspherin^®^. They appreciated clear and supportive communication from their doctors but found the written mandatory study information overwhelming. Despite pain, fatigue, and long recovery times after CRS-HIPEC, they remained optimistic and focused on returning to a meaningful life. Hope, trust in healthcare providers, and good communication played important roles in patients’ decisions to join this clinical trial.

## 1. Introduction

Peritoneal metastasis (PM) from colorectal cancer (CRC) is associated with a poor prognosis and a short life expectancy (6–12 months) if left untreated [1]. In cases with a limited tumor load of PM, patients may gain improved survival following a combination of complete cytoreductive surgery and hyperthermic intraperitoneal chemotherapy (CRS-HIPEC) [2]. However, the outcome of CRS-HIPEC remains variable. Indeed, many patients will experience disease recurrence, either as visceral metastases to the liver or lungs, as metastases to the lymph nodes, as a new peritoneal recurrence, or a combination of these. In one study, the five-year overall survival (OS) was 36%, whereas the corresponding five-year disease-free survival (DFS) was only 14% [3]. Patients with either primary or recurrent PM face the most dismal prognosis [4], which illustrates the need for improved treatment [5]. Attempts to improve outcomes by adding early postoperative intraperitoneal chemotherapy have shown promising disease outcome results in retrospective series but with significant added toxicity, and sound evidence from prospective randomized studies is still lacking [6,7,8,9].

Alpha-emitting radiopharmaceuticals have gained increasing attention in oncology due to their unique radiobiological properties. Alpha particles have a very short tissue penetration range, typically less than 100 μm, combined with a high linear energy transfer. This results in highly localized cytotoxic effects capable of inducing complex and irreparable DNA damage, while largely sparing surrounding healthy tissue.

In the context of intraperitoneal disease, these properties are particularly advantageous. After cytoreductive surgery, residual tumor burden often consists of microscopic tumor deposits on peritoneal surfaces or free-floating cancer cells. Intraperitoneal administration of α-emitting agents allows direct irradiation of these surfaces, offering a biologically compelling approach for eliminating residual disease while minimizing systemic toxicity.

Radspherin^®^ is an investigational product in clinical development designed to administer local, short-range, ionizing radiation to the surface of body cavities. It consists of bio-degradable carbonate microparticles that have absorbed the α-emitting radionuclide ^224^Ra. Via bolus injection into the peritoneal cavity two days after the completion of the established standard treatment CRS-HIPEC, the particles are compartmentally distributed in the abdominal cavity. Emitted internal α-particle radiation to the peritoneal surfaces will potentially kill any remaining small tumor cell clusters and free-floating cancer cells [10,11,12]. The aim is to prevent subsequent peritoneal disease recurrence.

The results of a phase-1 dose-escalation study with Radspherin^®^ administered intraperitoneally in subjects with PM from CRC following CRS-HIPEC have established the recommended dose, a good safety and tolerability profile, as well as a promising sign of clinical efficacy [13,14]. From July 2022, an open phase-2a expansion cohort recruited 17 patients from Norwegian Radium Hospital, Oslo University Hospital (NRH OUH), who received a single postoperative administration of Radspherin^®^ at the recommended activity dose of 7 MBq. Ten of the 17 patients constitute the patient population in the present study.

Patients’ global, physical, and functional quality of life (QoL) typically declines after CRS-HIPEC but gradually returns to baseline within 6–12 months, with long-term survivors often showing sustained improvement [15,16]. Social QoL also tends to be reduced, though less consistently. Lower QoL has been associated with older age, female gender, prolonged surgery, extensive or residual disease, adjuvant chemotherapy, postoperative complications, stoma formation, and disease recurrence–particularly early recurrence, which negatively impacts QoL at both 6 and 12 months [17,18]. Qualitative studies further highlight how postoperative chemotherapy, treatment side effects, and limited access to coordinated rehabilitation and healthcare services negatively affect patients’ well-being and underscore the need for stronger support networks for patients and their families [19,20,21].

While the clinical development of novel intraperitoneal radionuclide therapies such as Radspherin^®^ primarily focuses on safety, tolerability, and oncological outcomes, little is known about how patients experience participation in these early-phase trials. In particular, patients’ expectations, hopes, and interpretations of receiving an experimental α-emitting treatment in the immediate aftermath of extensive surgery remain unexplored. Understanding these experiences is essential for ethical trial conduct, informed consent, and patient-centered follow-up care.

The aim of this qualitative study was to explore participants’ experiences of participating in an adjuvant clinical trial with Radspherin^®^ as the intervention, with CRS-HIPEC providing a contextual background.

## 2. Materials and Methods

In line with the study’s objectives, research questions, and psychosocial orientation, we adopted a qualitative design informed by phenomenological and interpretive traditions. A phenomenological orientation emphasizes how illness and treatment are experienced and understood by individuals in their everyday lives, drawing attention to the embodied and emotional dimensions of recovery [22]. The interpretive (hermeneutic) perspective recognizes that understanding develops through interaction between participants and researchers, shaped by their preconceptions and the broader clinical and social context. Meaning is therefore viewed as co-constructed rather than objectively discovered [23,24].

### 2.1. Participants

Patients with PM from CRC treated at the NRH OUH and enrolled in a phase-1/2a study (EudraCT 2018-002803-33, ClinicalTrials.gov NCT03732781) to evaluate the efficacy of Radspherin^®^ were included in this research project. More specifically, this qualitative study aimed to recruit 15 participants from the NRH OUH. Due to the gap in the timeline from the start of the clinical phase 2a study to the point of obtaining the mandatory approvals for this qualitative study, as well as certain logistical challenges, not all eligible patients could be included. Three patients were not available for interview. Two eligible patients declined participation because they felt too unwell at the time of recruitment. Consequently, the final sample consisted of ten participants.

Key inclusion criteria were histologically confirmed colorectal cancer with peritoneal metastases, eligibility for complete cytoreductive surgery with HIPEC, and postoperative administration of intraperitoneal Radspherin^®^. Participants were required to be 18 years of age or older and able to provide informed consent. Exclusion criteria followed the clinical trial protocol and included conditions precluding CRS-HIPEC, significant comorbidities, or contraindications to intraperitoneal radionuclide therapy. No additional inclusion or exclusion criteria were applied specifically for the qualitative study.

Five men and five women participated in this study, all of white Western ethnicity. The patients’ median age was 63 years (range: 42–71 years) at the time of surgery.

Guided by the concept of information power [25], the limited sample size was considered adequate given the focused study aim, the high specificity of the sample (CRC with PM undergoing CRS-HIPEC followed by postoperative intraperitoneal Radspherin^®^), the richness afforded by two interview rounds, and an analytic strategy prioritizing depth and interpretive understanding. Nevertheless, we acknowledge that not reaching the planned sample size may have reduced the likelihood of capturing the full range of perspectives.

Characteristics of the participants are shown in Table 1. In Section 3 of this paper, as well as in Table 1, the participants are identified as P1–P10. The initial round of 10 interviews was conducted 8 to14 days after the patients had completed treatment with CRS-HIPEC and Radspherin^®^ at NRH OUH, each lasting 34–60 min. The second round included nine interviews during the follow-up period, typically 10–12 months post-treatment, conducted either at NRH OUH (*n* = 3) or by telephone (*n* = 6), each lasting 32–54 min.

Participant P2, who rapidly developed liver and lymph node metastases, had by then succumbed to the disease. Seven participants had synchronous PM, while the remaining three had metachronous PM after a median disease-free interval of 14 months (range: 11–21). Selected clinico-pathological parameters, collected from medical records as specified in the study-specific case report forms, are presented in Table 1.

### 2.2. Procedure

The participants in the clinical trial were identified and recruited by the last author, SGL, the principal investigator (PI) of the phase 1/2a clinical study. In this qualitative study the nurse A.H., handled all contact, provided information and recruited the participants. Recruitment to the qualitative study followed a consecutive approach and was determined by participation in the ongoing phase 1/2a Radspherin^®^ clinical trial. All participants treated at NRH OUH who met the inclusion criteria for the trial were invited to participate, and ten agreed to take part in this sub-study. Because the qualitative project was nested within the clinical protocol, the research team did not influence sample composition beyond these clinical inclusion parameters. The resulting group included variation in age, gender, and disease characteristics, providing a meaningful basis for exploring shared and differing experiences across participants.

Individuals willing to consider participation received a study information letter regarding possible recruitment. It was made clear that participation was entirely voluntary and that the patients could withdraw from the study at any time without providing a reason. Moreover, it was explained that participation in the study would have no implications for the standard treatment or scheduled follow-up visits in the ongoing clinical phase1/2a study. Written informed consent was obtained from all participants who agreed to participate in the study.

The first author, LF, conducted all the interviews. The interviews were transcribed verbatim by a medical secretary at the NRH OUH. Two rounds of interviews were conducted to capture participants’ experiences at different stages of the treatment trajectory. The first interview took place shortly after CRS-HIPEC and Radspherin^®^ administration and focused on immediate experiences related to diagnosis, decision-making, information provision, and the acute postoperative period. The second interview was conducted during follow-up and enabled participants to reflect on how their understanding of and expectations related to trial participation developed over time, recovery, and longer-term physical and psychosocial consequences. The interview guide is presented as Supplementary Material (File S1).

In their first interview, the participants were invited to briefly narrate their experiences from the time of diagnosis to the present day. They were asked about their experiences of receiving information related to participation in the Radspherin^®^ study and their reasons for agreeing to participate. Additionally, the participants were questioned about their experiences of Radspherin^®^ treatment during the postoperative period. In the second interview, the focus shifted, and the topics discussed included patients’ experiences of long-term side effects deemed related either to CRS-HIPEC or Radspherin^®^, potential recurrence or metastasis, new treatments, and the impacts on patients’ social, vocational, physical, and existential life. Furthermore, the information and follow-up that the patients had received were also explored. Finally, we asked them to convey their thoughts about the future.

While the interviews mainly focused on experiences related to participation in the Radspherin^®^ trial, participants’ accounts naturally encompassed the broader treatment trajectory, including CRS-HIPEC, which formed the clinical context for trial participation.

The longitudinal interview approach enabled exploration of change and continuity in participants’ experiences over time. By revisiting participants during follow-up, the study captured how initial expectations, interpretations, and emotional responses were reconsidered and reinterpreted in light of recovery, ongoing challenges, and uncertainty about the future.

### 2.3. Ethical Considerations

In accordance with the principles of ICH-GCP 2.1 to 2.3, the foreseeable risks, inconveniences, and patient rights associated with this study have been considered. Approval to conduct interviews and to collect, as well as store sensitive data, was obtained from the data protection officers at the NRH OUH (2019/4479185) and from the Regional Committee for Medical Research Ethics (2018/2313). All gathered data were stored confidentially, while the interview transcripts were de-identified. To ensure rigor of the study, the Consolidated Criteria for Reporting Qualitative Studies (COREQ) was followed [26], and the checklist is available as Supplementary Material (File S2). The participants are identified as P1–P10 to protect patients’ confidentiality.

### 2.4. Analysis

We conducted a reflexive thematic analysis, following Braun and Clarke’s guidelines for systematically and transparently identifying, analyzing, and reporting patterned meanings across qualitative data [27,28]. The analytic process involved (1) familiarization with the data through repeated reading of the transcripts, (2) generation of initial codes capturing both semantic and latent meanings, (3) searching for patterns and potential themes across codes, (4) reviewing and refining themes in relation to the coded data and the full dataset, (5) defining and naming themes, and (6) producing. The analysis was inductive and data-driven, aiming to remain close to participants’ own language and experiences while allowing for interpretative depth. All interviews were conducted and transcribed in Norwegian. The analysis was carried out in the original language to retain the nuances of participants’ expressions and contextual meanings. The first author immersed herself in the data by repeatedly reading the interview transcripts and conducting detailed, line-by-line coding. This initial coding phase was conducted manually by LF and involved generating analytic labels that captured both semantic (explicit) and latent (underlying) meanings within participants’ accounts.

Codes were then reviewed, compared, and collated into preliminary categories that reflect shared meanings or conceptual connections. These categories were further refined into themes and overarching concepts through an iterative and interpretative process guided by continuous reflexivity [27]. As a research team, we engaged in multiple analytical discussions to explore how different patterns of meaning related to one another, to challenge individual interpretations, and to ensure that the developing themes remained coherent and grounded in the data. See Table 2.

Illustrative quotations representing the diversity and richness of participants’ perspectives were systematically identified and selected to exemplify the analytical insights. Throughout the process, we repeatedly revisited the original transcripts to verify the consistency of each theme and to ensure that participants’ voices and intentions were preserved during interpretation and translation [29]. The final themes were conceptualized not as objective findings, but as interpretative stories about participants’ experiences, co-constructed through reflexive engagement between the researchers and the data.

### 2.5. Reflexivity and Researcher Positioning

Reflexivity was addressed through ongoing analytic discussions within the multidisciplinary team, where assumptions related to clinical roles, expectations of novel treatments, and the trial context were critically examined. Rather than attempting to eliminate contextual influences, these were treated as integral to understanding how participants made sense of their experiences in an early-phase oncology trial.

In addition, recruitment from the same institution where participants received treatment may introduce social desirability or gratitude-related bias, whereby participants feel inclined to emphasize favorable experiences. To mitigate such influences, all interviews were conducted by a qualitative researcher who was neither involved in participants’ clinical care nor follow-up. Participants were explicitly informed that participation was voluntary and would not affect their treatment. While these measures likely reduced the impact of contextual bias, they could not fully eliminate the influence of the clinical trial setting on participants’ accounts. The findings should therefore be understood as context-dependent and interpretive, reflecting how participants made sense of trial participation within a highly specialized and trust-based clinical environment.

The research team represented a combination of clinical and psychosocial perspectives, which allowed for both insider and outsider understandings of the study context. The first author (LF), a qualitative researcher with a background in psychosocial oncology, conducted all interviews and led the analysis. This distance facilitated open dialogue with participants while still enabling an informed understanding of the medical setting. The clinical co-authors (SGL, ØSB, AH) contributed contextual and interpretive insights during analysis, helping to ensure clinical accuracy and relevance. Throughout the research process, reflexive discussions were used to examine how the researchers’ professional backgrounds, expectations, and interpretations might influence data collection and analysis.

## 3. Results

Through the thematic analysis, four interrelated themes were identified, reflecting the participants’ experiences throughout the Radspherin^®^ study and their broader treatment trajectory: (1) receiving information about the clinical trial, (2) sustaining hope for cure, (3) managing challenges related to CRS-HIPEC, and (4) experiences of follow-up care.

### 3.1. Navigating Complex Information Through Trust and Dialogue

Participants described the written study information as extensive, formal, and difficult to absorb, particularly given their physical and emotional vulnerability before surgery. The 11-page information sheet, received after a phone conversation with the PI, contained all required details on the experimental treatment, side effects, ethics, and data protection. While participants recognized the necessity of these details, several found the material overwhelming and too complex for their condition at the time.

“It was very heavy and difficult reading. […] No, I wouldn’t say that I didn’t understand it, but I spent a lot of time thinking it over, read it several times. It could have been written much more simply.”

(P9)

Some needed help reading or interpreting the text, as many described how nausea, pain, and exhaustion made it difficult to focus.

“Yes, I managed to read everything, but I don’t know if it all registered. Maybe some of it slipped my mind because I was sick, in pain, and nauseous. But I did the best I could.”

(P4)

Participants emphasized that verbal communication with the PI and other healthcare professionals was clear, supportive, and decisive for understanding the trial and feeling safe in their decision to participate. They appreciated the time given for questions and the honest tone, with no unrealistic expectations being raised.

“I feel I’ve received very good information the whole way through. I’m very focused on trusting those who have the expertise we need. […] It was that openness and the conversations we had, the right talks, without him promising me anything at all.”

(P3)

Most patients agreed readily to participate, motivated by trust in their doctors and confidence in the research. P5 initially declined participation due to fear of radiation exposure but later changed his mind after receiving further information. P1 expressed hesitation about potential side effects, and P2 found the idea somewhat frightening, but both ultimately decided to participate.

“Radioactive radiation in the body… I didn’t understand that […] and I didn’t want to be a guinea pig. But once I talked to them […] and they explained, then I said yes.”

(P5)

### 3.2. Hope, Trust, and Altruism as Motivational Forces

The hope that Radspherin^®^ might contribute to eliminating their cancer was the strongest motivation for participation. Other factors also played a role, notably that patients had been informed that previous clinical studies with Radspherin^®^ had shown promising results with minimal side effects, and that participation offered the prospect of closer follow-up through clinical examinations, blood tests, and computed tomography (CT) scans.

“I’m not sure I would’ve been cancer-free if I hadn’t been in that study. I believe it did something, […] I also feel that I’ve been better cared for than if I hadn’t participated. […] Of course, I have much more hope now after having Radspherin put into my abdomen. […] I hope I might get a few more years on this Earth.”

(P4)

Confidence in previous safety data reinforced their motivation to participate, especially for those who feared radiation-related side effects.

“The most important thing was that similar treatments had been performed for other similar patients before, and they hadn’t shown major side effects. I thought that was crucial to know because what you really fear are side effects. If it doesn’t work, that’s one thing, but side effects… that’s scary.”

(P1)

For several, the decision also reflected altruism—a wish to contribute to research and help future patients.

“If it could help others after me, then I said yes, so that those doing research on this will have more to build on. That’s why I chose to take part.”

(P5)

Radspherin^®^ was administered two days after CRS-HIPEC, when participants were still in pain and extremely weak. The procedure itself went smoothly, though the necessary repositioning of the body was described as unpleasant. This was a mandatory procedure believed to improve the peritoneal distribution of the agent.

“Two days after the operation, they had to turn me from side to side, let me lie there for half an hour. That was somewhat painful so soon after surgery.”

(P2)

Despite uncertainty about its actual therapeutic effect, participants reported no side effects they attributed to Radspherin^®^. Many associated study participation with hope, safety, and close medical attention.

“Everything was fantastic with the follow-up from the Radium Hospital. Maybe it was because I was in the Radspherin study; I had check-ups every three months where they did CT scans and all kinds of examinations. […] I didn’t notice any difference. Everything is okay, no problems because of Radspherin; it seems to be helping. You can see that I’m here and still healthy.”

(P7)

Even those who experienced recurrence expressed no regret about joining the study and said they would have participated again.

“I’ve been thinking that it might not have worked so well for me, since I got a recurrence in the abdominal cavity. But these things happen, […] I would have joined [the study] again. […] I believe in research; I would have agreed to it. You can’t have bad luck all the time.”

(P8)

Hope was intertwined with gratitude, trust, and altruism. Participants viewed Radspherin^®^ as both a medical opportunity and a symbolic expression of faith in science and recovery, helping them sustain optimism through a difficult postoperative course.

### 3.3. Enduring the Physical and Emotional Aftermath of Major Surgery

Recovery following CRS-HIPEC was described as physically and mentally exhausting. All participants experienced substantial pain, fatigue, nausea, and functional limitations in the weeks after surgery. Stoma management, infections, and reduced appetite were common challenges, as well as concerns about managing daily life and family responsibilities after discharge.

“It was a very tough and major operation, with open surgery and the HIPEC they put in, which made me very sick. […] I was in very bad shape when I woke up, so it’s been really tough, and I’ve now been here for two weeks. […] I think it’s going to be hard to go home, both physically and mentally. I have three children, and it will be tough with all the noise and all the things going on.”

(P1)

Although satisfied with hospital care, several noted limited nutritional guidance and lack of clarity about long-term prognosis. Functional recovery often took months, and some required further chemotherapy or surgery due to recurrence. Persistent challenges included fatigue, bowel dysfunction, sleep disturbances, hormonal changes, anxiety, and fear of recurrence.

“I had to use a wheelchair and couldn’t walk from the kitchen to the living room when I got home. […] About a month after I got home from the Radium Hospital, I developed an infection. […] It took several months before I could walk 100 m. It was very frustrating and tough.”

(P3)

One participant experienced a serious late complication requiring emergency reoperation:

“In January [surgery was mid-November], I was at home sitting on the toilet, feeling very nauseous, and then I felt my whole body shaking. […] It ended with me undergoing another ten-hour surgery since they found a bowel perforation. […] I left the hospital in early March and was placed in a nursing home, mostly filled with 90-year-olds. […] I needed help with medication, help getting up, help going to the bathroom… a nightmare.”

(P10)

Over time, some regained strength and resumed part-time work, supported by rehabilitation and physiotherapy, while others continued to struggle with fatigue and emotional distress.

“I prioritized my rehabilitation, which has been fantastic, with all the help I received there and with a physiotherapist to build myself back up. Today, I’m in better shape than I was before the surgery. It’s been a tough time, but I’ve seen progress and set myself a goal. I fought hard, and things have gone well.”

(P3)

Persistent fear of recurrence was common and often intensified before follow-up scans.

“I’ve changed, of course. […] I can’t work the way I used to, but otherwise, we try to live as normally as possible. […] I don’t think too far ahead anymore. I assume I won’t get very old, but I just take it one day at a time and try to make the best of it.”

(P8)

Participants’ accounts reveal the profound physical and psychosocial burden of CRS-HIPEC, with recovery shaped by pain, fatigue, and fear of recurrence. Supportive rehabilitation and personal motivation were key to regaining function and coping emotionally.

### 3.4. Balancing Reassurance and Unmet Needs in Follow-Up Care

Participants valued the structured follow-up after discharge—initially every three months, then every six—because regular scans and consultations provided reassurance.

“Yes, I’ve had very close follow-up, which has been good. At the same time, there are some things I wonder about regarding the treatment that they don’t have answers for, especially concerning pelvic issues and severe diarrhea. […] That part has been difficult, but otherwise the follow-up is very thorough.”

(P1)

However, many described unmet needs related to long-term side effects, psychosocial support, and uncertainty about the role and potential impact of Radspherin^®^ after the acute phase

“Lots of information before the operation. […] But after that, they haven’t mentioned a word about Radspherin. They don’t follow up that part at all. There’s no talk about what that Radspherin stuff does, or has done, or should have done. […] It’s simply not a topic.”

(P10)

Participants appreciated medical surveillance but wished for more guidance on late effects, rehabilitation, and everyday challenges after treatment. Follow-up care was experienced as medically thorough but limited in addressing long-term physical and psychosocial needs. Participants expressed a desire for more continuity and open dialogue about both recovery and the potential impact of Radspherin^®^.

Across all themes, participants’ experiences reflected a tension between vulnerability and resilience. Hope, trust in clinicians, and a willingness to contribute to research were central to how they made sense of participating in an early-phase clinical trial. At the same time, the lasting physical burden of CRS-HIPEC and the emotional uncertainty of recovery underscored the need for clear communication and long-term support.

## 4. Discussion

While the immediate postoperative period was characterized by pain, complications, fatigue, and a demanding daily life, many participants articulated a pronounced sense of hope. Patients were motivated both by the possibility of cure and by the prospect of an added survival benefit associated with Radspherin^®^ without any radiation-related side effects.

They emphasized that information regarding participation in the clinical trial should be tailored to those with a severe disease burden who are scheduled for advanced combined treatment comprising CRS-HIPEC and Radspherin^®^. Furthermore, they expressed the need for sufficient information about potential side effects and long-term sequelae.

The participants described the 11-page information sheet in advance of the Radspherin^®^ treatment as being too long and complex. Some were initially skeptical of the treatment, especially regarding the “radiation exposure” and its potential side effects. As reported by others, further discussion with their physician eased their concerns [30]. Our findings suggest that written materials should be better adapted to patients in a vulnerable situation due to advanced cancer. Many have undergone previous treatments and are about to go through a major surgical procedure. In line with other studies [30,31], several participants expressed that helping future patients was a reason to participate in this clinical trial, even if the doctors could not guarantee a positive health-related outcome.

Options to further improve clinical outcomes following CRS-HIPEC in CRC patients with PM are sorely needed. The treatment goal is to preserve the “surgical complete response”, and the several available treatment options have recently been reviewed [5]. Sequential postoperative intraperitoneal chemotherapy holds promise [32]. In contrast to Radspherin^®^ given just once and directly after CRS-HIPEC, such chemotherapy is administered repeatedly over 6 months with significant added toxicity and subjective burden for the patients.

The interviews clearly confirmed that the side effects of CRS-HIPEC, as well as forsome a disease recurrence, contributed to the participants experiencing a challenging life. The long-term complication rate following CRS-HIPEC has been described in a large population-based cohort from Sweden [33], and the experiences among our participants are in line with this. Importantly, none of the participants reported any of the complications deemed related to Radspherin^®^. In one patient (P10), however, a possible relationship between the experienced complications and the ionizing radiation from Radspherin^®^ could not be ruled out. He underwent an emergency laparotomy for a small bowel perforation 72 days after CRS-HIPEC and Radspherin^®^. A late perforation after CRS-HIPEC alone is a known but rare condition, occurring in approximately 1% of cases [33]. The α-particles emitted from ^224^Ra during decay have a very short irradiating range, mostly affecting just the serosal linings of the peritoneal cavity, and do not penetrate deeper into the intestinal wall [34]. This strongly contrasts with colloidal particle therapies based on ^32^P chromic phosphate microparticles that emit energetic beta-radiation with a maximum range of several millimeters in tissues. These therapies were used for decades and were reported to be as effective as adjuvant cisplatin in patients with PM from ovarian cancer [35]. However, they were later abandoned due to the high rate of late bowel complications. A similar situation has also been reported for the widely studied and previously used partial abdominal external beam radiotherapy [36,37].

The development of Radspherin^®^ should also be viewed in the broader context of the rapidly evolving field of radionuclide-based targeted cancer therapy. Radiopharmaceuticals using α- and β-emitting isotopes are emerging as promising treatment modalities across several tumor types. They offer highly localized cytotoxic effects with minimal systemic toxicity.

Advances in radiopharmaceutical delivery and dosimetry are expected to expand their therapeutic role in oncology over the coming years [38,39]. As such, clinical trials investigating new agents like Radspherin^®^ are likely to become more common, emphasizing the need to understand not only safety and efficacy, but also how patients experience and interpret these novel therapies.

This study adds to the existing literature an in-depth understanding of how patients experience participation in an early-phase intraperitoneal radionuclide trial in the immediate aftermath of CRS-HIPEC. While previous research has described quality-of-life trajectories and postoperative challenges, little is known about how patients interpret and make sense of a novel adjuvant treatment during a highly vulnerable postoperative period. By exploring expectations, hope and trust in this specific context, the study offers insights that may inform both future trial design and patient communication strategies.

A dominant finding of this study was that all participants emphasized hope for an improved outcome or even a cure, combined with reassurance from frequent medical follow-ups, as central to their decision to participate. Despite postoperative challenges and, for some, disease recurrence, none expressed regret about joining the trial. Consistent with previous research, hope functioned as a vital psychological resource for coping with uncertainty and illness [40]. Participation in the clinical trial appeared to strengthen this hope. It was reinforced both by the belief in a new treatment and by the closer follow-up, which made participants feel safe and cared for.

Such perceptions are well documented in early-phase oncology trials, where hope and potential therapeutic benefit may coexist. Hope in this context should not be understood solely as a misunderstanding of trial purpose, but also as a psychological and existential resource that enables patients to cope with uncertainty and severe illness. At the same time, therapeutic misconception raises important ethical considerations, underscoring the need for communication strategies that balance optimism with realistic expectations.

Moreover, hope in this context extended beyond the prospect of cure to include a wish to regain normality, fulfill family roles, and plan for the future despite uncertainty [41]. Many described balancing gratitude and optimism with awareness of possible early death. For some, focusing on the present offered emotional stability, while others continued to struggle with lasting complications and fear of recurrence, consistent with earlier findings [42].

Previous studies suggest that hope can be fostered through therapeutic communication and psychosocial support [40,43]. Yet, physicians do not always recognize its significance [44]. When healthcare professionals focus solely on medical facts, they may unintentionally diminish hope and thereby weaken patients’ trust. Developing a more nuanced understanding of hope, as both a coping mechanism and a relational process, may help clinicians address the psychological and social dimensions of cancer care [45].

Our findings show that serious diagnoses and postoperative challenges affect patients and heighten family anxiety and children’s emotional distress. They may also hinder social and occupational reintegration. Consistent with earlier research findings, these observations affirm that psycho-oncological interventions, such as family counseling and workplace accommodations, play a crucial role during the extensive convalescence period [16]. In line with other studies, our participants experienced a demanding daily life and unmet needs [15,16,17]. Developing comprehensive strategies to minimize suffering and provide a higher level of support should remain a central focus of both research and clinical care for patients with peritoneal surface malignancies [46].

Although participants consistently described the clinical follow-up as close and reassuring, some emphasized that study-specific communication about Radspherin^®^ itself was “not a topic” during later consultations. A possible implication of these findings is the need for more structured opportunities to address trial-specific questions during follow-up. Integrating brief, intentional check-ins about the experimental component at selected follow-up visits may help clarify expectations, reduce uncertainty, and support patients’ sense of coherence. Patients valued being informed not only about their overall progress but also about the treatment component that had motivated their participation.

An additional ethical consideration is how patients in early-phase oncology trials balance uncertainty about benefit with strong hope for improvement. This highlights the need for transparent communication that supports realistic expectations while acknowledging the role of trust and altruism in decision-making. Ensuring this balance is essential to supporting patients throughout their participation.

A notable strength of this study is its two-round interview process, which captured both short- and longer-term challenges and allowed the participants sufficient time to reflect on those experiences. This study included a small number of participants from a single institution, which limits the transferability of the findings. In addition, the relatively homogenous patient group reflects the specific clinical context of a single tertiary center, and the findings should therefore be understood as context-specific rather than broadly generalizable. One participant died before the second interview. This loss inevitably reduced variation in the follow-up data and may have introduced a survivorship bias.

Given the clinical context and the limited pool of eligible participants, only ten patients were available for inclusion. We therefore could not pursue full data saturation in a traditional sense. However, during analysis, we observed a high degree of thematic convergence across participants, with no substantially new concepts emerging in the later interviews. We acknowledge that the small sample imposes a methodological limitation and that additional participants might have generated further nuance. This constraint reflects the realities of recruitment within a clinical trial rather than a lack of analytic rigor.

The use of various interview modes in the second interview round represents a potential methodological limitation. Telephone interviews may reduce access to non-verbal cues and embodied expressions of distress, fatigue, or uncertainty, potentially affecting the depth and nuance of the data. As a result, some experiential aspects, particularly those related to bodily vulnerability and emotional intensity, may have been less richly articulated than in face-to-face interviews. At the same time, telephone interviews may have facilitated participation and openness for some participants by reducing burden and allowing conversations to take place in familiar surroundings. Although consistent interviewing procedures were applied across modes, interview mode may have shaped how experiences were expressed and should be considered when interpreting the findings.

The second round of interviews was conducted 10–12 months after treatment, a period chosen to move beyond the acute postoperative phase and capture experiences related to longer-term recovery, reintegration into everyday life, and reflections on trial participation. However, this timeframe may not fully capture late-onset effects or further evolution in perspectives over time. Future qualitative studies with extended follow-up periods, such as 24 months or longer, may provide additional insights into survivorship challenges, late effects, and ongoing meaning-making processes following CRS-HIPEC and experimental intraperitoneal treatments.

Because this qualitative study was nested within an early-phase clinical trial, participants are likely to represent a subgroup characterized by relatively high trust in clinicians, strong hope for benefit, and a positive orientation toward research participation. Such contextual factors may influence how experiences are interpreted and narrated, potentially amplifying positive accounts of trial participation and limiting the expression of critique or ambivalence. Although interviews were conducted by a researcher not involved in clinical care and participants were informed that their responses would not affect treatment, the institutional and trial context may nonetheless have shaped participants’ accounts and should be considered when interpreting the findings. In addition, the predominantly positive narratives and the absence of more critical reflections may partly reflect dynamics commonly seen in early-phase oncology trials. Hope, trust, and altruism can shape how patients make sense of their experiences, and a wish to support research or to be perceived as cooperative may influence how openly concerns are expressed. These contextual factors do not undermine the findings but highlight the complexity of interpreting patient accounts in experimental treatment settings. As this was a qualitative study focusing on patients’ lived experiences, the findings should not be interpreted as evidence of treatment efficacy or causal relationships. Participants’ perceptions of benefit or improvement reflect their subjective interpretations rather than objective clinical outcomes. The study provides insight into how patients understood and interpreted receiving Radspherin^®^ in the context of extensive surgery and recovery, but it cannot determine whether these experiences were directly caused by the intervention itself.

The sample size was small and drawn from a single tertiary-care institution, and all participants were of white Western ethnicity. While this homogeneity reflects the specific clinical context, it may limit the transferability of the findings to more diverse, multicultural settings. As a qualitative study, the findings are not intended to be statistically generalizable but to provide in-depth, context-sensitive insights into how patients experience participation in an early-phase intraperitoneal clinical trial.

## 5. Conclusions

Despite undergoing advanced surgery, experiencing postoperative challenges, and suffering fear of recurrence, the participants emphasized hope for a better outcome as a key motivation to participate in the clinical trial. Clear oral communication from physicians and close clinical follow-up contributed to patients’ sense of safety and trust, although some noted that Radspherin^®^ was not sufficiently discussed after the acute treatment phase.

As new treatments such as Radspherin^®^ are further developed, it is essential to continue examining the patient’s perspective to ensure that they receive thorough information and support throughout the process while maintaining their hope in a balanced and realistic way.

## 6. Clinical and Research Implications

The findings underscore the importance of integrating psychosocial and communicative perspectives into early-phase oncology trials. Patients’ willingness to participate was shaped not only by clinical information but by how hope, trust, and meaning were conveyed. Tailored communication that balances realism with optimism may strengthen informed consent and enhance patients’ sense of safety. Clinicians should recognize that follow-up care after CRS-HIPEC involves both medical surveillance and the need for emotional processing of an intensive treatment experience.

Future early-phase trials may benefit from repeated, phase-specific discussions that clearly distinguish between safety-focused trial objectives and potential but uncertain therapeutic benefit. Integrating explicit opportunities to revisit expectations during follow-up may help support informed participation while preserving patients’ sense of hope and trust.

For future trials involving novel intraperitoneal agents such as Radspherin^®^, incorporating patient-reported experiences as secondary endpoints could improve understanding of treatment acceptability and perceived benefit. Developing structured pathways for psychosocial support and study-specific communication throughout follow-up may reduce uncertainty and unmet needs. Finally, these insights can inform the design of patient information materials, ensuring they are concise, accessible, and attuned to the vulnerability of individuals undergoing complex multimodal cancer treatment.

## Figures and Tables

**Table 1:** Selected clinico-pathological characteristics.

P	T	N	Lnmet	M	SM	DFI	Chemo Before	PCI	MMC	Knife Time	ANA	SAE	Dis	ACC	Chemo After
P1	T3	N2a	5/22	M1c	S	0	Yes	8	61	550	1	1	16	2	No
P2	T4a	N2b	16/18	M1c	S	0	Yes	11	69	426	1	2	10	2	No
P3	T3	N1b	2/12	M1c	S	0	No	8	70	468	1	1	13	2	No
P4	T4a	N0	0/23	M1c	M	14	Yes	11	62	418	0	1	28	3	No
P5	T4b	N1b	2/29	M1c	S	0	Yes	2	68	345	1	0	10	2	Yes
P6	T4b	N1b	3/28	M1c	S	0	No	8	62	337	1	0	14	2	Yes
P7	T4a	N1a	1/13	M1c	S	0	No	5	70	330	1	1	15	2	Yes
P8	T3	N1a	1/29	M1c	M	21	Yes	14	70	464	3	0	15	2	Yes
P9	T4b	N2b	6/15	M1c	S	0	No	7	64	386	1	0	9	2	Yes
P10	T3	N1b	2/16	M1c	M	11	Yes	8	70	509	1	0	12	3	No

Abbreviations: P: patient number; TNM: stage of tumor, node, and metastasis; Lnmet: number of lymph nodes with metastases and harvested; SM: synchronous or metachronous peritoneal metastases; DFI: disease-free interval before peritoneal metastases; Chemo Before: chemotherapy for the disease prior to CRS-HIPEC; PCI: peritoneal cancer index; MMC: mg of mitomycin C given as HIPEC; Knife Time: operation time in minutes; ANA: number of bowel anastomoses; SAE: severe adverse events; Dis: discharged from hospital (days postoperatively); ACC: Accordion score; Chemo After: chemotherapy after CRS-HIPEC.

**Table 2:** Overview of themes, descriptions, and illustrative codes.

Overarching Theme	Brief Description	Illustrative Codes
Navigating Complex Information Through Trust and Dialogue	Participants experienced the written study information as extensive, formal, and difficult to absorb due to physical and emotional vulnerability before surgery. Understanding and reassurance were primarily established through trust-based dialogue with clinicians rather than written material alone.	Overwhelming amount of information; Information received at a vulnerable time; Difficulty concentrating due to pain, nausea, and fatigue; Needing help to read or interpret information; Multiple information documents at once; Fear of side effects; Fear of radiation; Clear and honest verbal explanations; Not being promised benefit; Trust in the principal investigator; Openness and transparency; Feeling guided rather than persuaded
Hope, Trust, and Altruism as Motivational Forces	Hope for cure or prolonged survival was the strongest motivation for participation and was closely linked to trust in clinicians, confidence in prior research, and the perception of closer medical follow-up. Altruism and the wish to contribute to future treatment options also shaped participants’ decisions.	Hope of being cancer-free; Hope of long-term survival; Fear of recurrence; Balancing hope and uncertainty; Trust in medical expertise; Confidence in previous safety data; Closer follow-up as reassurance; Willingness to participate despite uncertainty; Altruistic motivation; Desire to help future patients; Feeling fortunate to be included in the study
Enduring the Physical and Emotional Aftermath of Major Surgery	CRS-HIPEC was described as an extremely demanding treatment with profound physical and psychological consequences. While Radspherin^®^ itself was not perceived to cause specific side effects, uncertainty remained regarding its role and effectiveness within the overall treatment trajectory.	Major and invasive surgery; Severe postoperative pain; Extreme fatigue and physical exhaustion; Anxiety and emotional distress; Stoma-related challenges; Infections and postoperative complications; Difficulty distinguishing treatment-related symptoms; Physical discomfort during repositioning; Limited expectations due to early-phase study; Enduring treatment without knowing the outcome
Balancing Reassurance and Unmet Needs in Follow-Up Care	Follow-up care was experienced as medically thorough and reassuring due to frequent imaging and consultations. However, participants reported unmet needs related to late effects, rehabilitation, psychosocial support, and limited information about Radspherin^®^ after treatment.	Regular follow-up scans as reassurance; Anxiety before follow-up appointments; Living with ongoing uncertainty; Lack of information about long-term effects; Absence of dialogue about Radspherin^®^ after treatment; Persistent fatigue and reduced functioning; Managing everyday life after discharge; Fear of recurrence resurfacing over time; Need for guidance and continuity of care; Taking life one day at a time

## Data Availability

The qualitative interview data contain potentially identifiable patient information and are therefore not publicly available, in accordance with Norwegian data protection regulations. De-identified excerpts relevant to the study’s findings are available from the corresponding author upon reasonable request and within the limits of participant confidentiality.

## Supplementary Materials

The following supporting information can be downloaded at: https://www.mdpi.com/article/10.3390/cancers18020244/s1, File S1: Interview Guide; File S2: Consolidated criteria for reporting qualitative studies (COREQ): 32-item checklist.
