# Supplementary material for: Experiences and Hopes Among Patients with Colorectal Carcinoma and Peritoneal Metastases Who Are Participating in an Early-Phase Clinical Trial
Source: Cancers (Basel). 2026 Jan 13;18(2):244. doi: 10.3390/cancers18020244 (PMC12838663; doi:10.3390/cancers18020244)
Supplement: Supplementary file 1 [file cancers-18-00244-s001.zip › cancers-4037338-supplementary File S1.pdf]

(File S1)

**Interview round**	**Thematic focus**	**Example interview questions**
Round 1 (Retrospective)	Treatment trajectory	*Can you tell me shortly about your experiences from the time you were diagnosed until now?*
Round 1 (postoperative)	Information and consent	*How did you experience the information you received about the Radspherin® study?*
Round 1 (postoperative)	Motivation and expectations	*What were your main reasons for deciding to participate in the Radspherin® clinical trial?*
Round 1 (postoperative)	Early experiences with treatment	*How did you experience receiving Radspherin® after surgery?*
Round 1 (postoperative)	Side effects and recovery	*Have you noticed any symptoms or side effects that you associate with the Radspherin® treatment?*
Round 2 (follow-up)	Long-term recovery	*How has your recovery been since you returned home after treatment?*
Round 2 (follow-up)	Physical and psychosocial consequences	*How has the CRS-HIPEC treatment affected your everyday life, work, or family situation?*
Round 2 (follow-up)	Meaning-making and reflection	*Looking back, how do you now think about your decision to participate in the clinical trial?*
Round 2 (follow-up)	Follow-up and support	*How have you experienced the follow-up care and information you have received over time?*
Round 2 (follow-up)	Future outlook	*What are your thoughts and hopes regarding the future?*
