# Supplementary material for: Experiences and Hopes Among Patients with Colorectal Carcinoma and Peritoneal Metastases Who Are Participating in an Early-Phase Clinical Trial
Source: Cancers (Basel). 2026 Jan 13;18(2):244. doi: 10.3390/cancers18020244 (PMC12838663; doi:10.3390/cancers18020244)
Supplement: Supplementary file 1 [file cancers-18-00244-s001.zip › cancers-4037338-supplementary File S2.pdf]

(File S2)

Consolidated criteria for reporting qualitative studies (COREQ): 32-item checklist

No	Item	Guide questions/description	Response
**Domain 1: Research team and reflexivity**			
Personal Characteristics			
1.	Interviewer/facilitator	Which author/s conducted the interview or focus group?	Lena Fauske conducted the interviews
2.	Credentials	What were the researcher's credentials? *E.g. PhD, MD*	LF: MA PhD<br>ØB: MD PhD<br>AH: RN<br>SGL: MD PhD
3.	Occupation	What was their occupation at the time of the study?	LF: Researcher<br>ØB: Oncologist, professor<br>AH: Study nurse<br>SGL: Gastro surgeon
4.	Gender	Was the researcher male or female?	Two were female and two were male
5.	Experience and training	What experience or training did the researcher have?	LF: Qualitative research/patients perspective, medical humanities<br>ØB: Clinical and translational research in oncology<br>SGL: Surgical research<br>LF/ØB/SGL: Are all experienced counsellors for several master/PhD-students

No	Item	Guide questions/description	Response
Relationship with participants			
6.	Relationship established	Was a relationship established prior to study commencement?	Not by LF and ØB<br>Yes, SGL and AH
7.	Participant knowledge of the interviewer	What did the participants know about the researcher? e.g. *personal goals, reasons for doing the research*	The participants were told about the interviewers' professional background, and the reason for conducting the study, that she was not a health-care professional.
8.	Interviewer characteristics	What characteristics were reported about the interviewer/facilitator? e.g. *Bias, assumptions, reasons and interests in the research topic*	Experienced qualitative researcher<br>Well known with the cancer patients perspective
**Domain 2: study design**			
Theoretical framework			
9.	Methodological orientation and Theory	What methodological orientation was stated to underpin the study? *e.g. grounded theory, discourse analysis, ethnography, phenomenology, content analysis*	An explorative qualitative research design with a reflexive and interpretive phenomenological approach, and thematic analyzes underpinned the study.
Participant selection			

No	Item	Guide questions/description	Response
10.	Sampling	How were participants selected? *e.g. purposive, convenience, consecutive, snowball*	10 of 15 available patients enrolled in the main clinical study were chronologically included.
11.	Method of approach	How were participants approached? E.*g. face-to-face, telephone, mail, email*	Face-to-Face<br>)
12.	Sample size	How many participants were in the study?	Interview one: 10<br>Interview two: 9
13.	Non-participation	How many people refused to participate or dropped out? Reasons?	One did not participate in interview two due to death
Setting			
14.	Setting of data collection	Where was the data collected? e.*g. home, clinic, workplace*	Interview one: all at the clinic<br>Interview two: Three at the clinic and six by phone
15.	Presence of non-participants	Was anyone else present besides the participants and researchers?	No
16.	Description of sample	What are the important characteristics of the sample? *e.g. demographic data, date*	The study included five men and five women. The patients' median age was 63 years (range: 42–71 years)
Data collection			
17.	Interview guide	Were questions, prompts, guides provided by the authors? Was it pilot tested?	The interview guide was drawn on the researchers previous experiences with research on cancer patients,

No	Item	Guide questions/description	Response
			published literature in the field, and clinical experience<br>No pilot
18.	Repeat interviews	Were repeat interviews carried out? If yes, how many?	Yes, two rounds of interviews
19.	Audio/visual recording	Did the research use audio or visual recording to collect the data?	All interviews were audiotaped and transcribed by a medical secretary
20.	Field notes	Were field notes made during and/or after the interview or focus group?	Yes, field notes were made after the interviews
21.	Duration	What was the duration of the interviews or focus group?	First interview 34–60 minutes<br>Second interview 32-54 minutes
22.	Data saturation	Was data saturation discussed?	No, because recruitment occurred within a clinical trial, only ten eligible patients were available. Full saturation could therefore not be pursued. Nonetheless, thematic convergence was observed, and no new concepts emerged in later interviews. We acknowledge this as a methodological limitation linked to the trial context.
23.	Transcripts returned	Were transcripts returned to participants for comment and/or correction?	No

No	Item	Guide questions/description	Response
**Domain 3: analysis and findings**z			
Data analysis			
24.	Number of data coders	How many data coders coded the data?	One, LF
25.	Description of the coding tree	Did authors provide a description of the coding tree?	Yes
26.	Derivation of themes	Were themes identified in advance or derived from the data?	Themes were derived from the data
27.	Software	What software, if applicable, was used to manage the data?	No software used
28.	Participant checking	Did participants provide feedback on the findings?	No
Reporting			
29.	Quotations presented	Were participant quotations presented to illustrate the themes / findings? Was each quotation identified? e.*g. participant number*	Yes, participant quotations were presented to illustrate the themes. A participant ID was assigned to each participant to ensure their confidentiality. Neither gender nor age was linked to the quotes for the same reason.
30.	Data and findings consistent	Was there consistency between the data	There was consistency between the data, but also deviations within the material

No	Item	Guide questions/description	Response
		presented and the findings?	which gave us rich and nuanced examples suitable to illuminate the experiences from the participants' own perspectives
31.	Clarity of major themes	Were major themes clearly presented in the findings?	Yes, five major themes were presented
32.	Clarity of minor themes	Is there a description of diverse cases or discussion of minor themes?	Yes, deviations within the material were presented
